# Human Blood Extracellular Vesicles Activate Transcription of NF-kB-Dependent Genes in A549 Lung Adenocarcinoma Cells

**DOI:** 10.3390/cimb44120411

**Published:** 2022-11-30

**Authors:** Yulya I. Savinovskaya, Anna A. Nushtaeva, Anna V. Savelyeva, Vitaliy V. Morozov, Elena I. Ryabchikova, Elena V. Kuligina, Vladimir A. Richter, Dmitriy V. Semenov

**Affiliations:** Institute of Chemical Biology and Fundamental Medicine, Siberian Branch, Russian Academy of Sciences, Lavrentiev Avenue, 8, 630090 Novosibirsk, Russia

**Keywords:** human blood extracellular vesicles, transcriptome, extracellular RNA, next-generation sequencing, A549 cells, NF-кB signaling

## Abstract

Extracellular vesicles (EVs) produced by various cell types are heterogeneous in size and composition. Changes in the RNA sets of EVs in biological fluids are considered the basis for the development of new approaches to minimally invasive diagnostics and the therapy of human diseases. In this study, EVs were obtained from the blood of healthy donors by centrifugation, followed by ultracentrifugation. It was shown that EVs consist of several populations including small exosome-like vesicles and larger microvesicle-like particles. The composition of EVs’ RNAs was determined. A549 lung adenocarcinoma cells were incubated with EV and the NGS analysis of differentially expressed genes was performed. During the incubation of A549 cells with EVs, the levels of mRNA encoding components for the NF-kB signaling pathway increased, as well as the expression of genes controlled by the NF-kB transcription factor. Overall, our results suggest that components of EVs trigger the NF-kB signaling cascade in A549 cells, leading to the transcription of genes including cytokines, adhesion molecules, cell cycle regulators, and cell survival factors. Our data provide insight into the interaction between blood EVs and human cells and can be used for designing new tools for the diagnosis and treatment of human diseases.

## 1. Introduction

Extracellular vesicles (EVs) are produced by many cell types and circulate in biological fluids, including blood. EVs contain proteins, DNA, RNA, lipids, and metabolites that can bind to target cells and be internalized by recipient cells, causing an immune response, differentiation, modulating apoptosis, and other essential physiological processes [1].

Currently, there is no well-established consensus on specific parameters of EV subtypes. There are evolving recommendations for characterizing their subtypes, particularly in complex biological fluids. However, based on the mode of biogenesis and size, EVs are classified into three major classes: exosomes, microvesicles, and apoptotic bodies [1,2,3]. Exosomes represent the smallest subtype of EVs, from 30 to 100 nanometers in diameter, and are formed in several stages. First, the formation of the early endosome; second, the internal budding of the endosomal vesicle membrane, leading to the formation of multivesicular bodies (MVBs) containing intraluminal vesicles; third, the fusion of MVBs with the plasma membrane of the cell, and the release of the exosomes into the extracellular space. Shedding microvesicles (ectosomes) are membrane vesicles from 50 to 2000 nm in diameter. The main difference between microvesicles and exosomes is their formation on the cell’s outer membrane. In the first stage, the phospholipids of the plasma membrane are redistributed, including the exposure of phosphatidylserine to its outer layer, resulting in the formation of domains that promote the budding of a part of the cytoplasm surrounded by the cell plasma membrane. Then the “bud” is separated from the plasma membrane with the participation of the actin–myosin complex. Apoptotic bodies are parts of a cell containing fragments of chromatin and organelles and are enclosed in a membrane with a diameter of 50 to 5000 nm. Apoptotic bodies are formed during apoptosis, which is a kind of programmed cell death. Unlike exosomes and microvesicles, apoptotic bodies in their content are not a product of selective transport, but of randomly distributed portions of the cytoplasm adjacent to the membrane of the apoptotic cells [2,3].

It is considered that once released into the extracellular space, EVs can interact with target cell receptors. The ability to interact with cellular receptors allows EVs to address their contents toward specific cells, generating various biological responses in the recipient cells. Among ligand–receptor interactions, the ones related to toll-like receptors (TLR) should be of particular interest: TLR-2, TLR-4, and others [1,4].

In addition to mediating the intercellular exchange of information with their surface molecules, EVs are able to penetrate cells by macropinocytosis [5], endocytosis [6], and phagocytosis [7]. It is assumed that the contents of the EVs can enter directly into the cell by fusion of the plasma membrane with the outer membrane of the recipient cells [8]. EVs contain inflammatory proteins such as cytokines that regulate immune system response. Cytokines can reach the extracellular space packaged in EVs, facilitating the delivery and targeting of these molecules to distant target cells. Among cytokines, interleukins such as IL-1α, IL-1β, IL-6, IL-2, IL-4, and IL-33 are associated with the surface or are located inside the EVs [9]. Among the chemokines, CCL2, CXCL8, and others have been found to be associated with EVs. Various EVs also stimulate target cell activation through the TNFα signaling pathway. TNFα can stimulate the proliferation of normal cells, exhibit cytolytic or cytostatic activity against tumor cells, and induce inflammatory responses. With the growing number of studies on EVs, there is more and more evidence that EVs play an important role in the immune response, apoptosis, angiogenesis, and inflammation [1,4].

There is much direct and indirect evidence that EVs can deliver specific sets of RNAs to recipient cells, thereby participating in gene expression and epigenetic changes [10]. The transfer of microRNA and mRNA by EVs from one cell type to another and the functioning of the transferred RNA have been reported [11]. It is known that microRNAs of EVs are involved in the regulation of genes at the post-transcriptional level. The transfer of intact mRNAs with EVs from donor cells to recipient cells, in turn, can serve to exchange phenotypic traits between cells, since mRNAs of proteins enter the recipient cells, which initially were not expressed in them [12,13]. The public online database Vesiclepedia is a catalog of molecular data identified in all classes of EVs, including apoptotic bodies, exosomes, shedding microvesicles, and others [14]. Vesiclepedia currently contains over 27,000 mRNA entries and over 10,000 noncoding RNA entries. Many studies have focused on the different species of RNAs from EVs, including coding and noncoding species; the study of different transcript isoforms and splicing variants are results of the broad application of Rnaseq and computational tools [13,15]. However, the question remains as to exactly how RNAs and proteins in the composition of EVs influence the fundamental processes underlying intercellular communications [16].

In this work, an NGS analysis of RNAs obtained from human blood EVs was performed. The study revealed that EVs contain fragments of “housekeeping” rRNAs, tRNAs snRNAs, scRNAs, as well as the set of mRNAs encoding the major proteins in human blood cells (HBB, HBA2, ACTB, FTL, and others). We conducted a whole transcriptome analysis of gene expression changes in human A549 cells incubated with EVs from the blood of healthy donors. The EVs have been found to activate the NF-kB response, which in turn induces secondary changes—activation of genes involved in glycolysis, extracellular matrix assembly, regulation of cell adhesion, programmed cell death, and others. Data on components of blood EVs inducing the activation of the NF-kB signaling pathway indicate that EVs play an important role in modulating the immune response, inflammation, and apoptosis in target cells, and provide new insights into vesicle-mediated cell-to-cell communications.

## 2. Materials and Methods

### 2.1. Ethics Statement

Blood samples were obtained with informed consent from healthy donors in the Center of New Medical Technologies of ICBFM SB RAS (Novosibirsk, Russia). The study was approved by the Committee for Ethics of the ICBFM SB RAS (protocol number 9 of 7 September 2020).

### 2.2. Blood Donors

The study comprised healthy males and females aged 21–64 years with no clinical history of malignant neoplastic, autoimmune, or chronic inflammatory diseases.

### 2.3. Collection of Human Blood

Peripheral blood was obtained from the median cubital vein using BD vacutainer K3 EDTA (BD, USA). Samples were stored at +4 °C and subjected to fractionation no later than 2 h after collection.

### 2.4. Plasma EVs Isolation and Purification

EDTA-stabilized whole blood was centrifuged at 1200× *g*, for 20 min, at 4 °C. the blood plasma hemolysis index was estimated by the optical absorption of hemoglobin at 414 nm according to [17]. For further preparations, blood plasma samples with a hemolysis index lower than 2.0 were used. In order to remove cell debris and precipitated plasma proteins/lipids, blood plasma was centrifuged at 16,000× *g*, for 20 min, at 4 °C. Cell-free plasma samples were centrifuged at 160,000× *g* for 2 h at 4 °C. Obtained pellets were submitted to additional purification steps including three consecutive ultracentrifugations at 160,000× *g* for 2 h at 4 °C in an excess volume of Tris-buffered saline (TBS). Finally, each pellet containing the EVs was suspended in 100 μL of TBS, stored at −80 °C, and used for downstream applications. Further, the terms “EVs” and “EVs preparations” will be applied equivalently.

### 2.5. Dynamic Light Scattering Analysis (DLS)

Preparations of EVs were resuspended in a 1/100 volume of double-filtered phosphate-buffered saline (dfPBS). The size of the EVs was analyzed using a Malvern Zetasizer Nano ZS (Malvern Instruments Ltd., Malvern, UK), equipped with a 633 nm He–Ne laser, at 22 °C, and a detection angle of 173°. For each sample, 11 runs of 10 s were carried out three times. The received data were analyzed with Zetasizer software v7.11 (Malvern Instruments Ltd., UK).

### 2.6. Nanoparticle Tracking Analysis (NTA)

The concentration and size distribution profiles of EVs were determined using a NanoSight LM10 instrument (Malvern Panalytical, UK) equipped with a blue 488 nm laser light source. The samples were diluted 25 times in 0.22 μm dfPBS and mixed before being introduced into the sample chamber using a syringe pump with a fixed flow rate. The size distribution and particle concentration each represented the mean of three individual measurements with an acquisition time of 60 s, a camera level setting of 12, and a temperature of 25 °C. For video acquisition, a shutter setting was set to 600 and the sensitivity at 89 according to the system’s software guidance algorithms.

### 2.7. Transmission Electron Microscopy (TEM)

Samples of EVs (pellets) were fixed with 4% paraformaldehyde at 4 °C for 24 h, postfixed with 1% OsO_4_, dehydrated in graded ethanol and acetone, and embedded in an Araldite–epon mixture. Ultrathin sections (65–75 nm) from obtained hard blocks were prepared on an ultramicrotome EM UC7 (Leica, Wetzlar, Germany) using a diamond knife (Diatome, Nidau, Switzerland). Ultrathin sections were contrasted with water solutions of uranyl acetate and lead citrate and examined in a JEM 1400 TEM (JEOL, Tokyo, Japan). Digital images were collected using a Veleta side-mounted camera (EM SIS, Muenster, Germany).

### 2.8. Flow Cytometry Analysis

Preparations of EVs obtained at 160,000 g were stained with mouse anti-human CD3-FITC, CD79a-PerCP Cy5.5, CD41a-FITC, CD34-APC, and CD63-PE according to the manufacturer’s protocol (eBioscience, San Diego, CA, USA). Pellets were twice washed with dfPBS and analyzed using FACS Canto II (Becton Dickinson, Franklin Lakes, NJ, USA). Forward scatter and side scatter (FSC and SSC) PMT voltage settings were adjusted for the detection of extracellular particles (60–1000 nm) using CST beads (Becton Dickinson, USA) and 60 nm polystyrene beads (Thermo Scientific, Waltham, MA, USA). PMT voltage settings for the detection of FITC, PE, PerCP-Cy5.5, and APC fluorescence were adjusted using “Anti-Mouse Ig, *κ*/Negative Control Compensation Particles Set beads”, according to the manufacturer’s protocol (Becton Dickinson, Franklin Lakes, NJ, USA). The following settings were used for the flow cytometry analysis: FSC, 615; SSC, 310; FITC, 548; PE, 466; PerCP-Cy5.5, 505; APC, 300; threshold FSC/SSC, 200/200; compensation PE-FITC, 18; and compensation PerCP-Cy5.5/PE, 15. Gates were set according to unstained samples. Flow cytometry data were analyzed with BD FACSDiva software v6.1.3 (Becton Dickinson, USA). Overlaid histograms were created using Flowing software v2.5.1 (Turku University, Turku, Finland).

### 2.9. Cell Culture

The human A549 lung adenocarcinoma cell line was obtained from the Russian collection of cell cultures (Institute of Cytology of the RAS, Novosibirsk, Russia). The cells were cultivated in Dulbecco’s Modified Eagles Medium (DMEM) supplemented with 10% fetal bovine serum (FBS), 2 mM L-alanyl-L-glutamine, 100 U/mL penicillin, 0.1 mg/mL of streptomycin and 0.25 µg/mL of amphotericin B (Life Technologies, Carlsbad, CA, USA) at 37 °C in a humidified atmosphere containing 5% CO_2_.

### 2.10. Fluorescence Microscopy Analysis

Blood EVs were incubated with 1 mM fluorescein isothiocyanate (FITC) in 150 mM sodium bicarbonate buffer at pH 8.3 for 1 h at 25 °C. FITC-labeled vesicles were purified by three consecutive steps of centrifugation at 16,000× *g*, at 4 °C in TBS. The A549 cells were incubated with FITC-labeled extracellular vesicles for 24 h, fixed with formalin, stained with 4′,6-diamidino-2-phenylindole (DAPI), and analyzed with a ZOE Fluorescent Cell Imager (Bio-Rad, Hercules, CA, USA).

### 2.11. Viability of A549 Cells (MTT Assay)

The total protein concentration in EVs preparations was measured using a microvolume NanoVue Plus spectrophotometer (GE Healthcare, Chicago, IL, USA) and Qubit fluorimeter in combination with a Qubit Protein Assay Kit (Life Technologies, USA) following the manufacturer’s protocol. A549 lung adenocarcinoma cells were cultured as mentioned above, in 96-well plates, until a 40–50% confluence was reached. A549 cells were incubated with extracellular vesicle preparations (total EVs protein 10 ng/mL) for 48 h. MTT (3-(4,5-dimethylthiazol-2-yl)-2,5- diphenyltetrazolium bromide) (Applichem, Darmstadt, Germany) dye solution was added to the culture serum-free medium to a final concentration of 0.5 mg/mL and incubated for 4 h at 37 °C. The culture medium was removed, and the formazan crystals were dissolved with dimethyl sulfoxide (according to [18]). The absorbance at λ = 570 nm and background at λ = 620 nm was measured using a microplate reader (Apollo 8 LB 912, Berthold Technologies, Bad Wildbad, Germany).

### 2.12. Proapoptotic Changes of A549 Cells (FCM)

A549 cells were cultured, as mentioned above, in 6-well plates, treated with EV preparations, and incubated for 48 h at 37 °C. The cells were washed with PBS, and subsequently floating cells in the medium along with adherent cells were harvested by trypsin. After centrifugation at 800× *g* for 5 min, the cells were resuspended in 100 µL of 1 × Binding buffer. The solution was transferred to FACS tubes and 2 µL of propidium iodide (PI) and 4 µL of Annexin V-FITC conjugate were added according to the manufacturer’s protocol (BD-Pharmingen, USA). Finally, 300 µL of 1 × Binding buffer was added at room temperature, in the dark, for 15 min. Proapoptotic changes were determined by flow cytometry, using a BD FACSCanto II cytometer (BD Biosciences, Franklin Lakes, NJ, USA). The flow cytometry data were analyzed using FlowJo Software.

### 2.13. RNAs Extraction, cDNA Library Construction, Illumina Sequencing, and Differential Gene Expression Analysis

Total RNA was extracted from A549 cells with TRIzol Reagent (Life Technologies, USA) according to the manufacturer’s protocol. The RNA concentration was measured using a microvolume NanoVue Plus spectrophotometer (GE Healthcare, USA) and Qubit 2.0 Fluorimeter (Invintrogen, Waltham, MA, USA) with a Qubit RNA HS Assay Kit (Thermo Fisher Scientific, USA) following the manufacturer’s protocol. The quality of the total RNA as an RNA Integrity Number (RIN) was determined with a Bioanalyzer 2100 instrument (Agilent Technologies, Santa Clara, CA, USA) using an Agilent RNA Pico 6000 Kit (Agilent Technologies, USA). A threshold RIN reading greater than 8.0 was taken as the cutoff point for the transition to the stage of library preparation. The construction of the Illumina cDNA libraries and sequencing on the Illumina HiSeq 1500 was performed in Genoanalitica (Russia). Raw sequencing reads were controlled by FastQ analysis and subjected to adapter removal by Trimmomatic [19]. Trimmed sequencing reads were filtered with Bowtie2 using a reference containing sequences of rRNA, tRNA, snRNA, SINE, LINE, DNA repeats, and low complexity sequences, as well as mitochondrial DNA. Filtered reads were aligned and quantified with two independent approaches. The first approach used the human assembly GRCh37 (hg19) by HISAT2 using RefGene annotations to the human genome (https://hgdownload.cse.ucsc.edu/goldenPath/hg19/database/ accessed on 10 October 2020) [20]. The obtained data on the relative contribution of transcripts in the FPKM format were combined into cumulative tables using the CuffCompare from Cufflinks v.2.1.1 [21]. In the second approach, trimmed and filtered reads were mapped to the human genome (GRCh37/hg19) with STAR 2.7.1a [22], indexed with the RefGene human genome annotation. Aligned reads were quantified with QoRTs v1.3.6 [23], differential gene expression analysis was performed with DESeq2 [24], and R version 4.1.3, Bioconductor 3.14. The results of differential gene expression analysis—lists of up/downregulated genes—were interpreted with Enrichr [25] using the R interface to the Enrichr database.

### 2.14. Validation of mRNA Transcripts with qRT-PCR

A549 cells were incubated with EVs preparations for 24 h. Total RNA from A549 cells was isolated using TRIzol Reagent (Life Technologies, USA) with additional DNase I and RNase-free (Thermo Fisher Scientific, USA) digestion according to the manufacturer’s protocol. To determine the sensitivity of real-time RT-PCR, RNA was diluted to a concentration of 50 ng/µL. qRT-PCR was performed on a LightCycler 96 System (Roche, Basel, Switzerland), using the reagent kit BioMaster RT-PCR SYBR Blue (2×) (Biolabmix, Novosibirsk, Russia). The qRT-PCR conditions included the synthesis of cDNA at 45 °C for 30 min; initial activation at 95 °C for 5 min; 40 cycles with denaturation at 95 °C for 10 s, annealing at 58 °C for 10 s and extension step at 72 °C for 20 s, with a final extension at 82 °C for 5 s selected for primers GAPDH, NFKBIA, and CXCL1. The melting curves were analyzed to ensure the specificity of the products. The levels of mRNA were represented as relative values normalized to the level of GAPDH. To confirm the amplification of targeted gene fragments, PCR products were separated with electrophoresis in 1.5% agarose gel, stained with ethidium bromide, and documented with Gel Doc XR System (Bio-Rad, USA). The forward and reverse primers were synthesized in Biosset, Russia (Table 1).

## 3. Results

### 3.1. Isolation and Characterization of EVs from the Blood Plasma of Healthy Donors

**Figure 1:**
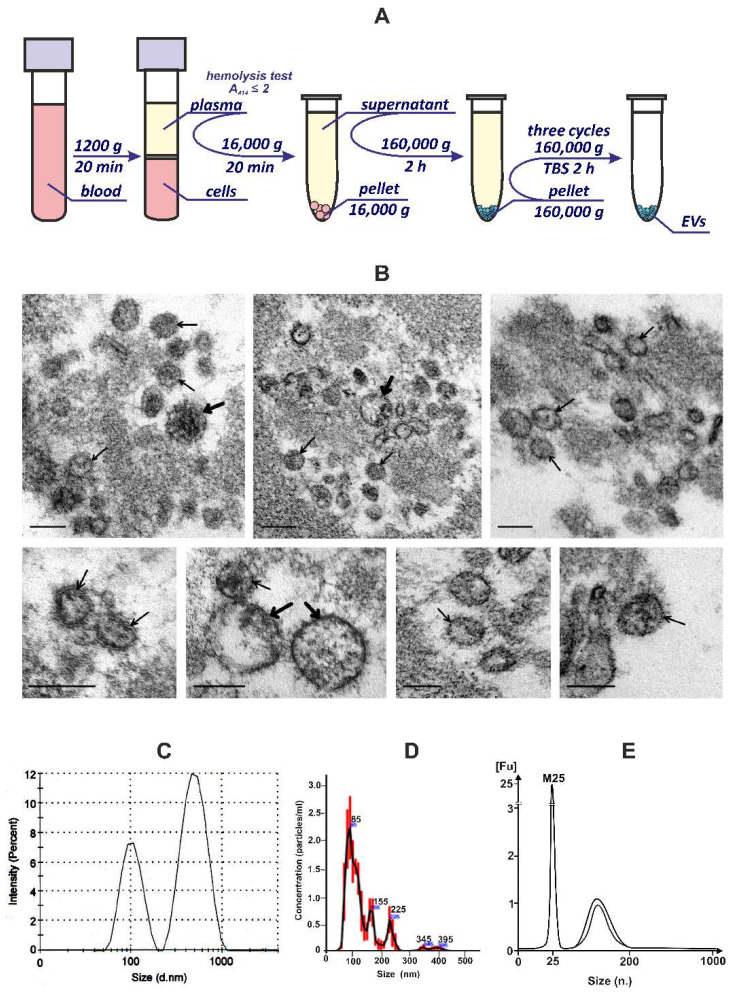
Human blood extracellular vesicle preparations. (**A**) Isolation and purification of extracellular vesicles from the blood of healthy donors. (**B**) Vesicles visualized by TEM in ultrathin sections of pellets (160,000× *g* ultracentrifugation). The upper row shows accumulations of vesicles, the lower row shows enlarged images of the vesicles. Thin arrows show vesicles with size and morphology corresponding to exosomes, and thick arrows, to microvesicles. Scale bars correspond to 100 nm. (**C**,**D**) The size distribution of extracellular vesicles was determined using dynamic light scattering and nanoparticle tracking analysis. (**E**) RNA length distribution of extracellular vesicles analyzed using Agilent Bioanalyzer 2100 on an RNA 6000 Pico chip; the RNA length marker (M25); n.—nucleotide.

The EVs of human blood are heterogeneous in size, origin, and molecular composition. Besides the EVs, human blood contains different non-vesicular macromolecular structures, for example, RNA–protein complexes and cellular components, that complicate the EVs isolation process [16,26]. To date, there is no standardized method for obtaining EV preparations [27]. The most frequently used methods for isolating EVs from blood plasma are based on ultracentrifugation [28]. In order to obtain the EVs from human blood, we used several consecutive centrifugation and ultracentrifugation steps that included low-speed centrifugation at 1200× *g* to remove blood cells; centrifugation at 16,000× *g* to remove cell debris and large particles followed by ultracentrifugation at 160,000× *g* to precipitate EVs (Figure 1A). We additionally purified the EVs pellets with three consecutive ultracentrifugations at 160,000× *g* to achieve EVs purification and preserve the RNA in the EVs preparations (Figure 1A).

The TEM study of ultrathin sections of the pellets (EVs preparations) revealed a fibrillar substance and various vesicles (30–200 nm in diameter), and single vesicles and their accumulations were observed (Figure 1B). Analysis of the obtained ultrastructural data confirmed that EVs preparations exactly contain vesicles; many of them corresponded to exosomes by size and spherical morphology. Usually, EVs are examined using negative staining; however, this method does not always reveal the membrane-limiting vesicles and, respectively, does not allow for the unambiguous identification of the detected structures. We have applied the ultrathin sections method that provides the accurate identification of all structures (Figure 1B), and, therefore, we can conclude that the preparations used in the work contain EVs and do not contain cellular organoids. Given that other methods in this study perceive vesicles as particles, both terms will be used the same way.

The EVs preparations contain particles with a hydrodynamic diameter of 50–200 and 300–1000 nm, according to DLS analysis (Figure 1C). According to the NTA analysis, the samples contained several subpopulations of EVs with a hydrodynamic diameter of ~85, 155, 255, and 350–400 nm, which correspond, by size, to exosomes, microvesicles, and apoptotic bodies. The largest contribution was represented by the subpopulation with an average size of ~85 nm (Figure 1D). The yield of purified EVs was 20–35 × 10^3^ particles/μL of blood plasma which is consistent with the published data [29,30].

Thus, the obtained data leads to the conclusion that blood EVs preparations (160,000 g fractions) contain vesicles up to 100 nm in diameter, which can be attributed to exosomes, as well as to larger particles, namely microvesicles. At the same time, the size distribution profiles between the microvesicles and exosomes are very similar, which also suggests that these two vesicle populations overlap in size [27].

It is known that EVs are produced by different types of cells [31]. The markers on the surface of the vesicle membranes are similar to those of the parent cell membrane. To analyze the protein markers of blood EVs and assess their cellular origin, flow cytometry was applied using the sets of antibodies to the following antigens: CD63—a transmembrane protein of exosomes/endosomes; T-cell antigen—CD3; B-cell antigen—CD79a; platelet antigen—CD41a. It has been established that a set of purified blood EVs obtained by ultracentrifugations at 160,000× *g* contains vesicles with a platelet/megakaryocyte marker (~43%), T-cell marker (~51%), and B-cell marker (~5%). Roughly ~50% of purified blood EVs carried exosome marker CD63 (Table 2).

Total RNA was isolated from preparations of EVs and the distribution of RNA lengths was analyzed by capillary electrophoresis using an Agilent Bioanalyzer 2100. It was found that the RNA of EVs is represented by a set of fragments with a length of ~30 to 100 nucleotides (Figure 1E).

### 3.2. Internalization of EVs by A549 Cells and Influence of Vesicles on the Cells’ Viability

**Figure 2:**
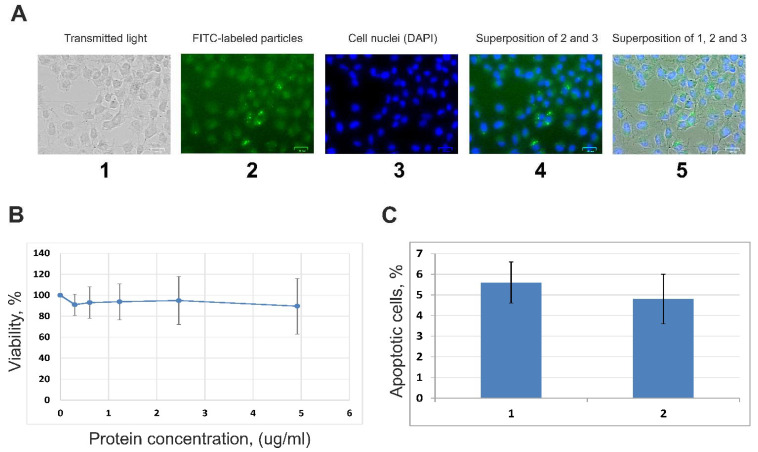
(**A**) Fluorescence microscopy of EVs internalization by human A549 cells. Cells were incubated with FITC-labeled human blood EVs for 24 h. 1—A549 cells in transmitted light; 2—fluorescence of FITC-labeled particles, 3—fluorescence of cell nuclei (DAPI); 4—superposition of 2 and 3; 5—superposition of 1, 2, and 3. Scale bars correspond to 25 µm (**B**). Viability of human lung adenocarcinoma A549 cells incubated in the presence of preparations of blood extracellular vesicles for 48 h (data of MTT-test). The curve corresponds to the change in the MTT index of cells during incubation with different concentrations of EVs. (**C**) Analysis of apoptotic changes in A549 cells under the influence of EVs from human blood by flow cytofluorimetry. The ordinate shows the contribution of cells stained with FITC-Annexin V and PI to the general population of A549 cells. Contribution of the apoptotic cells’ population to the total number of cells incubated for 48 h with (1) or without (2) preparations of blood EVs.

Using luminescence microscopy, it was found that after incubating A549 cells with FITC-labeled human blood EVs for 24 h, labeled structures were detected in the cytoplasm of recipient cells (Figure 2A). We determined that the EVs did not cause statistically significant changes in the A549 cells’ viability (Figure 2B) and did not induce noticeable proapoptotic changes in the cellular membrane as was assessed with flow cytometry (Figure 2C). Therefore, it can be concluded that the interaction of human blood EVs with A549 lung adenocarcinoma cells does not affect cell viability and cause apoptotic changes. At the same time, viable cells capture the components of EVs and accumulate them in the cytoplasm.

### 3.3. RNAs of Human Blood Extracellular Vesicles

To characterize the RNA profiles of EVs isolated from the blood plasma of healthy individuals, we used the Illumina RNAseq approach. EVs contain a significant number of fragments derived from rRNA covering up to 72% of all detected RNAs. The proportion of mRNAs, lncRNAs, and snoRNAs was about 25%. The proportion of reads annotated as small nuclear/cytoplasmic RNA including snRNA, scRNA, tRNA, and YRNA was, on average, 2 % (Table 3 and Table S1).

We detected the presence of small nucleolar RNAs (snoRNAs) and small Cajal bodies RNAs (scaRNA) in the set of EVs RNAs (Table S2). A number of nuclear lncRNAs—MALAT1, NEAT1, XIST, H19, MEG3, and TUG1—were also found (Table S3). Taken together, these data directly indicate the presence of nucleoplasm and nucleolus components in human blood EVs.

The majority of mRNAs in EVs preparations encode the cytoplasmic proteins of human blood cells: HBB, HBA2, FTL, and others (Table S4). From the results of Enrichr analysis, the set of 200 major mRNAs from EVs is enriched with RNAs, the transcription of which is controlled by transcription factors RELA, NFKB1, STAT3, and others (the library “TRRUST Transcription Factors 2019”, Table S5), and NELFE, CEBPD, MYC, and others (“ENCODE and ChEA Consensus TFs” from ChIP-X”, Table S5).

The mRNAs set of EVs preparations is enriched with transcripts encoding ribosome proteins, as well as focal adhesion and secretory granules proteins. In addition, the contribution of mRNAs encoding the proteins of circulating EVs including exosomes, and cellular organelles significantly increased in the set (“GO Cellular Component 2021” and “Jensen COMPARTMENTS”, Table S5).

200 major mRNAs of EVs encode proteins that participate in such biological processes as intracellular protein sorting; SRP-dependent cotranslational protein targeting to membrane; signal sequence recognition; double-stranded RNA binding; and rRNA binding, base pairing with RNA (“GO Biological Process 2021” and “GO Molecular Function 2021”, Table S5).

The mRNA set of EVs preparations is enriched with molecules characteristics for human blood cells and CD33+ myeloid cells (“Human Gene Atlas”, Table S5). These data suggest that myeloid cells make a significant contribution to the formation of a pool of human blood circulating EVs.

### 3.4. Gene Expression Changes in A549 Cells Treated with Blood EVs

In order to characterize cellular processes modulated by human blood EVs, we incubated lung adenocarcinoma A549 cells with preparations of EVs for 6, 12, and 24 h, then isolated total cell RNA and performed Rnaseq analysis using an Illumina HiSeq 1500. A total of 12 to 15 × 10^6^ experimental reads for each of the two biological replicates, for each of the three pairs of treatment/control (6, 12, and 24 h incubation of cells with EVs) were obtained.

**Figure 3:**
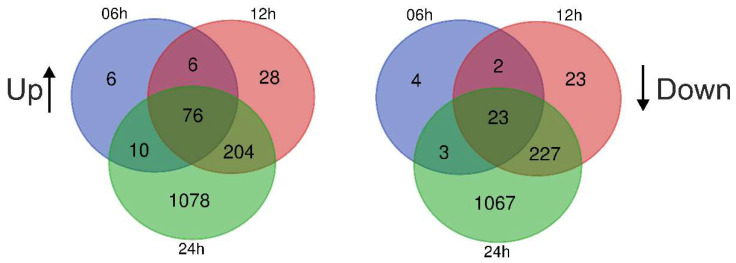
Venn diagrams showing intersections of differentially expressed genes in A549 cells that were incubated with human blood EVs for 6, 12, and 24 h. Up and Down—up- and downregulated genes.

**Figure 4:**
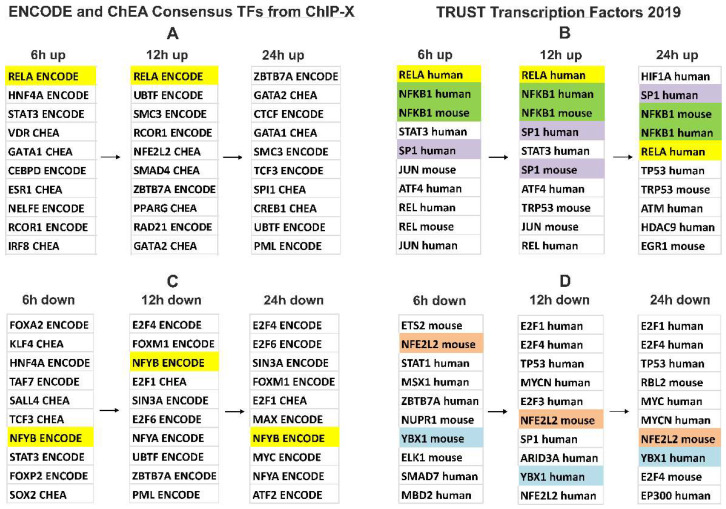
Transcription factors controlling gene expression changes in A549 cells incubated with human blood EVs according to the gene set enrichment analysis with Enrichr. (**A**,**B**)—factors involved in the control of upregulated genes. (**C**,**D**)—factors involved in the control of downregulated genes. Top 10 Enrichr records sorted by ascending *p*-value (Supplementary Tables S7–S12). Common top transcription factors for the differentially expressed gene sets (6, 12, 24 h; up or down) are highlighted in color.

It was found that the incubation of A549 cells with EVs preparations led to alterations in the expression of ~2200 genes. The levels of 76 mRNAs increased after 6 h of A549 incubation with EVs and remained constantly elevated after 12 and 24 h, while the levels of 23 mRNAs decreased after 6 h of incubation and remained decreased after 12 and 24 h (Figure 3). The genes of сhemokines, interleukin 32, TNFA, as well as NF-kB transcription factor subunits were the most significant representatives of early and constantly activated genes induced by EVs in A549 cells and, on the contrary, the expression of ATF5, CHD4, CNBP, and other genes was reduced (Table 4, Figure 4, Tables S7–S12).

The gene expression activation at the early stage (6 h) of cell incubation with EVs is controlled by the transcription factor RELA, belonging to the NF-kB family (Table S7, “TRRUST Transcription Factors 2019”). In addition, the products of upregulated genes interact and modulate the activity of the transcription factor NF-kB (Table S7, “KEGG 2021 Human”).

After 12 h of incubation of cells with vesicles, the expression of CXCL1, 2, 3, 5, and 8 was activated. Those genes are controlled by transcription factors RELA, NF-kB, SP1, and STAT3 (Table S8, “TRRUST Transcription Factors 2019”).

By 24 h of incubation of the A549 cells with EVs, the activation of transcription factors leads to generalized changes in the transcriptome which affects a series of large groups of genes, such as the chromatin assembly genes; chromosome segregation; DNA replication, and mitosis. Secondary response genes are controlled not only by NF-kB but also by several other transcription factors, including HIF1A, SP1, and TP53 (“TRRUST Transcription Factors 2019”, Table S9). These genes are involved in the TNF alpha-signaling via NF-kB, hypoxia, epithelial–mesenchymal transition (EMT), glycolysis, p53 pathway, and apoptosis (“MSigDB Hallmark 2020”, Table S9).

Thus, the response of A549 human lung adenocarcinoma cells to human blood EVs includes the activation of the transcription factor NF-kB at an early stage. This is followed by the activation of secondary transcription factors, including HIF1A, SP1, and TP53. The regulation of gene expression in the late stages of the action of vesicles on cells can include both NF-kB-dependent and independent processes (Tables S7–S12).

### 3.5. Verification of NGS Data with qRT-PCR

**Figure 5:**
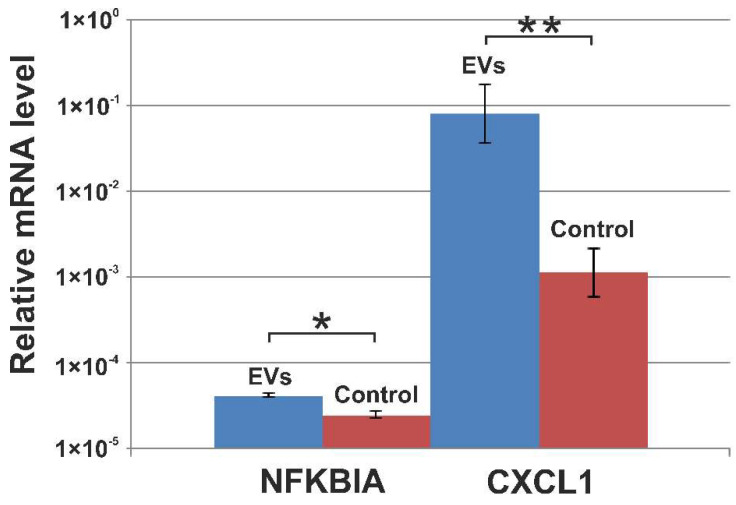
Relative NFKBIA and CXCL1 transcripts levels were evaluated by RT-qPCR following the incubation of A549 cells with EVs and in non-treated cells (Control). Cells were incubated with human blood EVs for 24 h. qPCR levels were normalized to the expression of GAPDH. The results are representative of four replicates (mean ± SD). * Indicates significant differences at *p* < 0.05 for NFKBIA and ** *p* < 0.01 for CXCL1 (Student’s *t*-test).

In order to confirm RNA sequencing data, we performed a qRT-PCR analysis of mRNAs NFKBIA and СХСL1, whose transcription is regulated by NF-kB. Relative transcript levels were analyzed using RNAs from A549 cells treated with EVs and non-treated cells. qRT-PCR data showed that EVs increased the expression level of the CXCL1 and NFKBIA genes in cells. Analyzed genes indicating significant differences at *p* < 0.05 (NFKBIA) and *p* < 0.01 (CXCL1) were determined (Figure 5). Thus, changes in the expression of two NF-kB-dependent indicator genes were in agreement with the RNA-seq results.

## 4. Discussion

The discovery of the RNA species diversity in EVs and the evidence that EVs are involved in a wide variety of physiological and pathophysiological processes has led to increased interest in EV RNA profiling. Currently, an increasing number of studies are aimed at profiling microRNAs [32,33,34,35] and mRNAs, but also a significant number of other species of vesicular RNAs, such as lncRNAs, rRNAs, tRNAs, circRNAs, snRNAs, snoRNAs, Y-RNAs, and others [36,37,38,39]. Huang X. et al. conducted sequence analysis for EVs RNAs obtained from blood, among which microRNAs were the most abundant and 76% of all mappable reads. Moreover, representatives of other RNA classes were detected, including rRNAs (9% of all mapped counts), lncRNAs (3%), piwi-interacting RNAs, tRNAs, and mRNA fragments (~1%), and snRNAs (0.1%) [40]. Amorim M.G. et al. found that 73% of the mapped reads corresponded to small noncoding RNAs, where tRNAs were dominant (57%), then mitochondrial rRNAs (15%), miscellaneous RNAs (misc-RNAs), and microRNAs (13%). Among the misc-RNAs were Y-RNAs (57%) and 7SL RNAs (40%) of the mapped reads followed by vault RNAs (3%), and 7SK RNAs (0.1%). The second most represented class was mRNAs (24%), followed by lnRNAs (2%), and pseudogenes (0.1%) [41]. Li Y. et al. sequenced the long RNA of EVs from blood and reported the distribution of RNAs by types in EVs: mRNA constituted 76% of the total mapped reads. Other RNA types accounted for a small fraction: 6% were circRNAs, 2% were pseudogenes, and 1% were lncRNA. Miscellaneous RNAs represented 15% of the total reads, including RN7SL1, RN7SL2, RN7SL4P, RN7SL5P, and the signal recognition particle (SRP) of metazoans. Many of the most abundant EV mRNAs are ribosomal protein-coding RNAs. The differentially expressed EVs RNAs participated in cancer development, including the influence on the adherent junction, HIF-1 signaling pathway, MAPK signaling pathway, and focal adhesion [42]. Recently, Rodosthenous R.S et al. showed a distribution of transcripts in EVs-enriched individual plasma, as well as long RNA species diversity: mRNA (9465), lncRNA (8110), pseudogenes (585), and ncRNA (376) transcripts per million kilobases (TPM). Significant pathways of genes include glucocorticoid biosynthesis, the intrinsic prothrombin activation pathway, multiple pathways related to thyroid hormone metabolism, extrinsic prothrombin activation pathway, eNOS signaling, and others [15].

In this work, we isolated and purified EVs from the blood of healthy donors by successive centrifugation and ultracentrifugation of blood plasma (Figure 1A). The analysis of human blood EV preparations carried out using TEM, DLS, and NTA showed that they contain vesicles, the sizes of which are comparable to those of exosomes and microvesicles (Figure 1). Using flow cytometry, we identified the protein markers of exosomes/endosomes: antigens of platelets, T, and B cells in obtained EVs preparations (Table 2).

In the NGS analysis of human blood EVs RNA, we detected fragments of rRNA (72%), small nuclear and small cytoplasmic RNAs (2.3%), mitochondrial transcripts (0.66%), and a set of mRNA and lncRNA (25%) (Table 3). The set of 200 most represented EV mRNAs was enriched with transcripts encoding ribosomal proteins, as well as proteins of focal adhesion, secretory granules, etc. Interestingly, the set was enriched with the mRNAs encoding components of exosomes and EVs (Table S5, “Jensen Compartments”). It can be assumed that EVs assembly occurs with the internalization of a part of the translation apparatus that produces the components of these membrane particles.

The set of 200 major mRNA of EVs was enriched with RNAs, the transcription of which is controlled by RELA and NFKB1 (Table S5, “TRRUST Transcription Factors 2019”). It can be supposed that in the cells producing the vesicles, both the NF-kB-dependent transcription and the NF-kB-signaling cascade were activated.

Based on available data, EVs may play a dual role in inflammation by stimulating proinflammation effects depending on the expression of surface markers and the microenvironment-affected cargo. It was found that EVs contain a variety of stimuli, including toll-like receptors ligands, proinflammatory cytokines such as interleukin IL-1, IL-6, and tumor necrosis factor-alpha (TNF-α), which promote the activation of nuclear factor kappa B (NF-kB). NF-kB represents a family of inducible transcription factors comprising five structurally related members (NF-kB1/p50, NF-kB2/p52, RelA/p65, RelB, and c-Rel), which regulate a large array of genes. These transcription factors are critical regulators of proinflammatory and stress-like responses that modulate the genetic programs sustaining cell growth, cancer cell survival, and cell motility, and regulating EMT and extracellular matrix homeostasis [43,44,45].

Recent studies demonstrate that EVs obtained from various sources can alter NF-kB activity. Schweiger M.W. et al. demonstrated that EVs originating from stem cell-derived glioblastomas can upregulate NF-kB and STAT3 through C/EBPβ, leading to increased aggressiveness and therapeutic resistance, features of the mesenchymal GBM subtype [46]. Wang B. et al. found that exosomes could deliver microRNAs-1910-3p to mammary epithelial cells and breast cancer cells, inhibiting MTMR3 expression, and activating the NF-kB and Wnt/β-catenin signaling pathway, thus promoting cancer cell proliferation, metastasis, and autophagy [47]. Jiannan Li et al. showed that microRNA-223 of platelet-derived exosomes inhibited the ICAM-1 expression in TNF-α-stimulated HUVECs cells through the regulation of the NF-kB and MAPK pathways [48].

In addition, Bretz N. P. et al. showed that EVs isolated from various body fluids could activate the NF-kB signaling pathway through TLR2 and TLR4 in monocyte cell models (THP-1 cells), leading to the release of various cytokines. TLRs are key receptors for the signaling from exosomes to cells. The analysis of the signaling pathways revealed an initial activation of NF-kB which induced the production of IL-6, required for further STAT3 activation [49]. Study Fabbri M. et al. found that microRNAs-21 and microRNAs-29a released by cancer cells in exosomes were binding to TLR7 and TLR8 to activate the NF-kB signaling pathway and may lead to tumor growth and metastasis [50]. Ye W et al. demonstrated that plasma-derived exosomes containing mtDNA can activate the NF-kB pathway in immune cells via TLR9 receptors, leading to the expression of proinflammatory genes [51]. Biemmi V. et al. demonstrated that plasma-derived EVs have a signaling capacity, interacting with TLR4 on the adult cardiomyocytes (CM) surface, activating the NF-kB signaling cascade that leads to the induction of apoptosis in CM [52].

**Figure 6:**
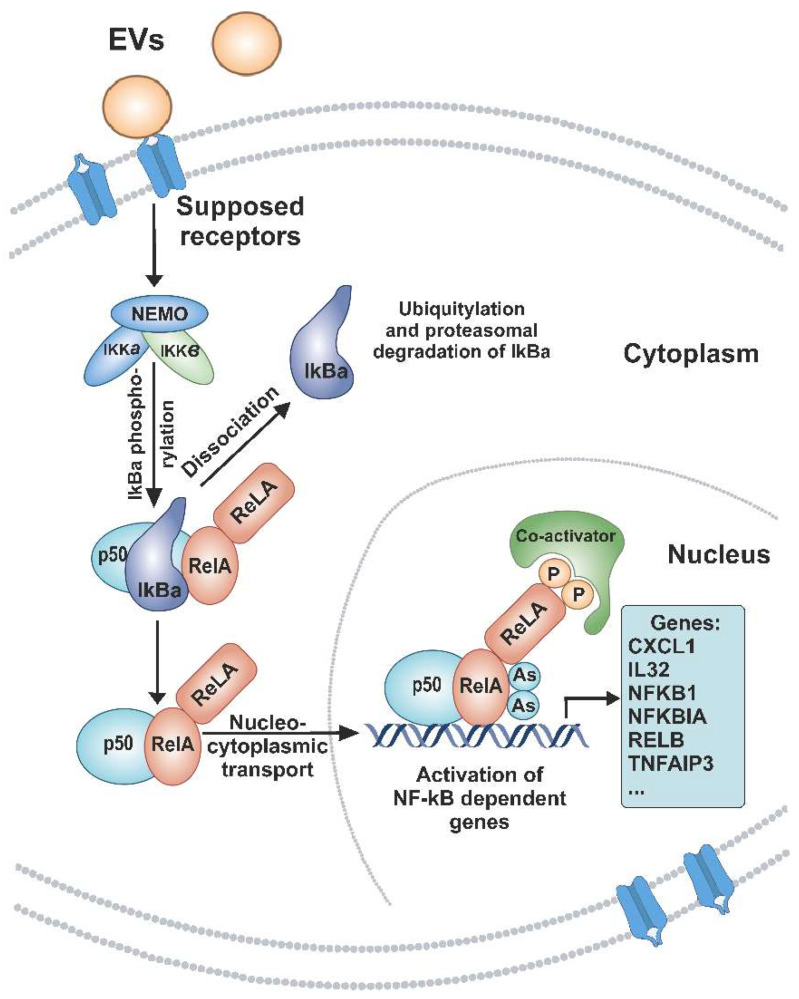
Hypothetical scheme summarizing the supposed processes of the NF-kB signaling pathway activation modulated by EVs and increased expression of mRNAs under the control of NF-kB. Extracellular vesicles interact with cellular receptors, which in turn, leads to a change in the activity of the kinase complex (IKK) with subsequent phosphorylation of IkBa. Phosphorylated IkBa dissociates, and undergoes ubiquitination and proteasomal degradation, which allows NF-kB dimers to move into the cell nucleus. This leads to NF-kB-dependent transcription activation.

Here we analyzed the influence of blood plasma EVs from healthy donors on gene expression in A549 lung adenocarcinoma cells. At an early stage (6 h) of incubation of A549 cells with EVs, the gene expression is controlled by the transcription factor RELA, which belongs to the NF-kB family. In addition, the products of these genes involved in the signaling cascade interact and modulate the activity of the transcription factor NF-kB (Table S7). It was also found that the level of 76 mRNAs increased after 6 h and remained increased after 12 and 24 h, while a level of 23 mRNAs decreased after 6 h and remained decreased after 12 and 24 h of incubation of A549 cells with EVs (Figure 3 and Table 3 and Tables S7–S12). As illustrated in Figure 6, our hypothetical model is that blood plasma-released EVs initiate a signaling cascade involving the supposed receptors and the activation of NF-kB in A549 cells. This leads to the fact that NF-kB activates the transcription of genes CXCL1, NFKBIA, and others (Table S6). Thus, our data indicate that the main result of the human blood EV influence on A549 cells is the fast and continuous activation of NF-kB-controlled transcription.

Secondary response genes, whose expression is activated by 24 h of incubation of A549 cells with EVs, are controlled not only by the transcription factor NF-kB but also by HIF1A, SP1, TP53, ATM, and others (Table S9). The products of these genes are involved in glycolysis, extracellular matrix assembly, the regulation of cell adhesion, and the regulation of programmed cell death processes. It allows for the suggestion that the activation of the NF-kB signaling pathway induces secondary metabolic and structural changes in target cells.

Thus, our data indicate that EVs, when interacting with human cells, activate the NF-kB signaling cascade and NF-kB-dependent transcription, which in turn modulates other downstream signaling and life processes.

## 5. Conclusions

In this work, we present the RNA composition of human blood EVs of healthy donors examined using an NGS approach. The study focused on describing the effect of human blood EV on processes in A549 human lung adenocarcinoma cells using the NGS analysis of gene expression changes. While human blood EVs themselves did not significantly affect the viability and apoptotic processes in A549 cells, the incubation of cells with EVs induced NF-kB activation at an early stage, and the variety of secondary processes controlled by transcription factors such as HIF1A, SP1, TP53, and ATM at later stages. The products of EVs-activated genes are involved in glycolysis, extracellular matrix assembly, the regulation of cell adhesion, and programmed cell death. It should be emphasized that the mRNA set of human blood EVs is also enriched in transcripts controlled by NF-kB. This allows for the proposal that the response of target cells partially reflects the processes occurring in cells releasing EVs. Overall, our data provide insight into the interaction between blood EV and human cells and can be used to develop new tools for diagnosing and treating human diseases.

## Figures and Tables

**Table 1:** Primer sequences used for qRT-PCR.

Gene	Sequences
GAPDH	F: GAAGGTGAAGGTCGGAGT
	R: GAAGATGGTGATGGGATTTC
NFKBIA	F: TGTCTACACTTAGCCTCTATC
	R: TCTGTGAACTCCGTGAACTC
CXCL1	F: AGGCAGGGGAATGTATGTGC
	R: AAGCCCCTTTGTTCTAAGCCA

**Table 2:** FCM analysis of protein markers in blood plasma EVs. The mean values and standard deviations of the contributions of individual fractions to extracellular vesicle preparations obtained from the blood plasma of three different donors are presented.

Markers	Content, %
CD63	51.4 ± 12
CD3	51 ± 22
СD79а	5.0 ± 3.5
CD41a	43.6 ± 11

**Table 3:** Contribution of the main classes of human RNAs to the total set of extracellular vesicle RNAs.

RNA Class	Reads × 10^−3^ (Ave)	Сontribution (%)
rRNA	2792	72
tRNA, U1-U12 snRNA, YRNA, scRNA, transcribed genomic repeats	90	2.33
Mitochondrial transcripts	25	0.66
Human genome transcripts (*hg19*) including RefGene RNA (mRNAs, lncRNAs, snoRNAs, and others)	968	25
Total	3875	100

**Table 4:** Representatives of genes whose mRNA levels were differentially expressed after 6 h as well as after 12 and 24 h of incubation of A549 cells with EVs.

Genes	Annotation
**Upregulated**	
CCL2	C-C motif chemokine ligand 2
CXCL1	C-X-C motif chemokine ligand 1
CXCL2	C-X-C motif chemokine ligand 2
CXCL3	C-X-C motif chemokine ligand 3
CXCL5	C-X-C motif chemokine ligand 5
CXCL8	C-X-C motif chemokine ligand 8
IL32	interleukin 32
NFKB1	nuclear factor kappa B subunit 1
NFKB2	nuclear factor kappa B subunit 2
NFKBIA	NFKB inhibitor alpha
RELA	RELA proto-oncogene, NF-kB subunit
RELB	RELB proto-oncogene, NF-kB subunit
RELT	RELT TNF receptor
TNFAIP1	TNF alpha-induced protein 1
TNFAIP2	TNF alpha-induced protein 2
TNFAIP3	TNF alpha-induced protein 3
TNFAIP8	TNF Alpha-induced protein 8
TNFRSF10B	TNF receptor superfamily member 10b
TNFRSF10D	TNF receptor superfamily member 10d
TNFRSF12A	TNF receptor superfamily member 12A
TNFRSF1A	TNF receptor superfamily member 1A
TNFRSF9	TNF receptor superfamily member 9
TNIP1	TNFAIP3 interacting protein 1
**Downregulated**	
ATF5	activating transcription factor 5
CHD4	chromodomain helicase DNA binding protein 4
CNBP	CCHC-type zinc finger nucleic acid binding protein
CSRP1	cysteine and glycine-rich protein 1
ETV4	variant transcription factor 4
KANK2	KN motif and ankyrin repeat domains 2
TRIM28	tripartite motif containing 28
UBTF	upstream binding transcription factor

## Data Availability

Not applicable.

## Supplementary Materials

The following supporting information can be downloaded at: https://www.mdpi.com/article/10.3390/cimb44120411/s1, Supplementary: Table S1: Мajor rRNA, snRNA, and scRNA of human blood EVs; Table S2: Мajor snoRNA and scaRNA of human blood EVs; Тable S3: Мajor lncRNA of human blood EVs; Table S4: Major mRNA of human blood EVs; Table S5: Analysis of properties of the 200 most represented mRNAs set of human blood EVs on Enrichr platform (http://amp.pharm.mssm.edu/Enrichr/ (accessed on 10 October 2021)); Table S6: Genes whose mRNA level changes through 6 h and persist by 12 and 24 h of incubation of A549 cells with EVs; Table S7: Characteristics of the genes whose mRNA level increases through 6 h of incubation of A549 cells with EVs; Table S8: Characteristics of the genes whose mRNA level increases through 12 h of incubation of A549 cells with EVs; Table S9: Characteristics of the genes whose mRNA level increases through 24 h of incubation of A549 cells with EVs; Table S10: Characteristics of the genes whose mRNA level decreases through 6 h of incubation of A549 cells with EVs; Table S11: Characteristics of the genes whose mRNA level decreases through 12 h of incubation of A549 cells with EVs; Table S12: Characteristics of the genes whose mRNA level decreases through 24 h of incubation of A549 cells with EVs.
